# How relevant is the mouse model for understanding human sex determination?

**DOI:** 10.7150/ijbs.90231

**Published:** 2025-10-15

**Authors:** Francisco Brito, Chloé Mayère, Aurélie Lardenois, Violaine Regard, Sylwia Czukiewska, Cyril Djari, Pauline Sararols, Yasmine Neirijnck, Françoise Kühne, Séverine Mazaud-Guittot, Susana M Chuva de Sousa Lopes, Frédéric Chalmel, Antoine D Rolland, Serge Nef

**Affiliations:** 1 Department of Genetic Medicine and Development, University of Geneva, 1211 Geneva, Switzerland.; 2 Univ Rennes, Inserm, EHESP, Irset (Institut de recherche en santé, environnement et travail) - UMR_S 1085, Rennes F-35000, France.; 3 Department of Anatomy and Embryology, Leiden University Medical Centre, Einthovenweg 20, 2333 ZC Leiden, The Netherlands.

**Keywords:** gonadal sex determination, single-cell transcriptomic, human, mouse, testis, ovary, Sertoli cells, pre-granulosa cells, germ cells, Leydig cells

## Abstract

The mouse is the most widely used model organism for studying mammalian gonadal sex determination and related human disorders. However, a systematic and comprehensive comparison of human and mouse sex determination processes is lacking. Here, we performed an interspecies comparative analysis of the single-cell transcriptomic atlas of gonadal sex determination in mice and humans. Our results revealed major transcriptomic differences in each of the major cell types between human and mouse gonads. Only a small fraction of these genes shared a comparable expression profile, often genes known to be essential for gonadal sex determination. While the most differentiated gonadal cell types share similar transcriptomic signatures between humans and mice, poorly differentiated cells, such as somatic progenitors, show more divergent profiles. Ultimately, these comparisons will identify the genes and pathways for which the mouse is a suitable model to study human gonadal abnormalities and optimise the use of animal models.

## Introduction

Gonadal sex determination is the process by which a bipotential gonad differentiates into a testis or an ovary. In vertebrates, both the cellular composition and morphogenesis of testes and ovaries are conserved. Indeed, in all clades, the gonadal primordium consists mainly of supporting cell precursors, steroidogenic precursors and primordial germ cells that commit to either an ovarian or testicular cell fate 1-4. In the developing testis, the supporting cell lineage differentiates into Sertoli cells (SCs), which enclose germ cells and form testis cords 5. The same SCs also secrete factors that promote the differentiation of the steroidogenic lineage into androgen-producing fetal Leydig cells 6-9. In the developing ovary, supporting cell progenitors form pre-granulosa cells that enclose primary oocytes to form follicles and promote the differentiation of steroidogenic progenitors into thecal cells 10-13. While the genetic and/or environmental signals that trigger gonadal sex differentiation into ovary or testis are not conserved in vertebrates - ranging from various environmental signals such as temperature to sex chromosome-related master genes - the genes or pathways involved in SC and pre-granulosa differentiation are generally well conserved 14, 15. These include the mutually antagonistic SOX9/FGF9 (Sertoli-testis) and RSPO1/WNT/β-catenin (pre-granulosa-ovary) pathways, which are widely conserved between mammals 16-19.

In humans, the majority of differences of sex development (DSDs) involving gonadal dysgenesis or aberrant testicular or ovarian determination remain unknown 20. Currently, our understanding of the signalling pathways and mechanisms that control sex-specific differentiation of the bipotential gonad in humans is largely derived from studies in the mouse model. In particular, the use of knock-out mouse models and advances in single-cell sequencing technologies have enabled a thorough investigation of the signalling pathways and mechanisms that control the gonadal differentiation process (reviewed in detail in 1, 21-25). It is generally accepted that these processes are largely conserved between mice and humans, implying that the mouse model is relevant for studying the process of human sex determination and providing information on the genetic aetiology of DSD cases. Many genes playing key roles in sex determination are conserved and have similar functions in these two species, including the essential and antagonistic roles of SOX proteins in testicular determination and the WNT signalling pathway in ovarian development 26. A number of genetic mutations leading to pathologies in humans have also been reported to disrupt sex determination in the mouse, confirming the similarity in the process of sex determination between these two species 27-42. However, there are also notable differences between human and mouse in the development of the gonads. For example, differences in their respective repertoires of regulatory elements 43 probably affect the spatiotemporal expression of key determining genes, such as *SRY*/*Sry*. In human XY embryos, *SRY* is expressed from E41 (around 6 weeks, just prior to sex determination), peaking soon after, and then decreasing but remaining stably lowly expressed until after embryogenesis 44. By contrast, in mice, *Sry* is only transiently expressed, over the sex determination period from E10.5 to E12.5 45. Another example is DMRT1 (Doublesex and Mab-3 Related Transcription factor 1) known to play an essential role in sex determination in many species, including fish 52, birds 53, 54 and several mammalian species, including goats 55 and rabbits 56. In humans, mutations affecting DMRT1 have been described in patients with 46,XY DSD with gonadal dysgenesis or ovotestis 32, 57, 58, suggesting that DMRT1 may be involved in testicular determination. The situation is quite different in mice, where DMRT1 does not appear to have retained a critical function in testicular determination, as targeted deletion of *Dmrt1* only affects postnatal testicular function and is required for testicular maintenance 59. Finally, the MAPK signalling is required for sex determination both in humans and mice. However, while gain-of-function mutations in *MAP3K1* have been shown to be associated with 46,XY complete gonadal dysgenesis in humans 60, loss of function of *Map3k4,* but not *Map3k1,* disrupts mouse testis determination 61, 62.

This raises the question of how similar the process of gonadal sex determination is between humans and mice. To our knowledge, there has never been a systematic and thorough analysis of interspecies differences in sex determination, particularly between humans and mice. In this study, we performed a comparative analysis of transcriptomic data at the single-cell resolution from human and mouse gonads during their differentiation. We compared the human and mouse transcriptomes for each of the major cell types of the developing testis and ovary, with a particular focus on the supporting-like and supporting lineages, as the specification of SCs or granulosa cells is the first crucial event in mammalian sex determination.

## Results

### Mouse and human single-cell transcriptomic atlases of gonadal sex determination

To systematically and comprehensively analyse the interspecies differences in the gonadal sex determination process between humans and mice, we used two large single-cell transcriptomic datasets covering the gonadal differentiation process in mice and humans. These two single-cell transcriptomic atlases are technically comparable, using a droplet-based 3′-end scRNA-seq (Chromium™ Single Cell 3' Library & Gel Bead Kit v2) from 10X Genomics with approximately 100,000 cells sequenced over a time course spanning the mouse (embryonic day (E)10.5-16.5) and human (post-conception weeks (PCW) 6-12) sex determination process.

To compare cell types and gene expression in the two species, we first analysed and annotated the datasets individually using an identical methodological approach (see Materials & Methods). The mouse single-cell transcriptomic atlas has been described and annotated previously in 63. It consists of 20 libraries from XX and XY gonads of mouse embryos at six developmental stages (E10.5, E11.5, E12.5, E13.5 and E16.5), covering the entire process of gonadal sex determination and the emergence and differentiation of the major testicular and ovarian lineages. The mouse dataset includes a total of 94,705 cells and 26,397 genes, with a median of 16,505 unique transcripts (UMIs) per cell, 4,341 genes per cell and 3.8 UMIs/gene per cell 63. The human dataset used in this study consists of 30 libraries (**Supplementary Figure S1A**), between 6 and 12 PCW, evenly split by sex per time point (15 XX, 15 XY), and was derived from a recently published study 64. It includes a total of 99,361 cells and 34,152 genes after filtering, with a median of 13,050 unique transcripts (UMIs) per cell, 3,429 genes per cell and 3.8 UMIs/gene per cell. To make the mouse and human datasets comparable, we re-analysed the human data using an identical approach to that used to analyse and annotate the mouse dataset. In short, the visualization of the single-cell transcriptomes using a uniform manifold approximation and projection space (UMAP) 65 shows that male and female human cells overlap at the earliest time point (6PCW), which progressively diverge over time (**Supplementary Figure S1B&C**). Leiden clustering resulted in a separation of the dataset into 41 distinct clusters (**Supplementary Figure S1D**). Differential expression analysis (DEA) was used to identify specific or enriched genes in each of the clusters (**Supplementary Table S1**), allowing us to manually assign a cell type to each of the 41 clusters. We annotated the 41 clusters into 15 distinct cell types (**Supplementary Figure S1D-F**): fetal Leydig cells, perivascular cells, steroidogenic progenitors, erythrocytes, germ cells, endothelial cells, immune cells, coelomic epithelial cells, mesonephros tubules, pre-supporting cells, female pre-supporting cells, pre-granulosa cells, male pre-supporting cells, SCs, and supporting-like cells (SLCs). The reannotated human data allowed to observe the same populations as in Lardenois *et al.* 64 showing the strong reproducibility of the results independently of the pre-analysis pipeline.

### Combined human and mouse single-cell transcriptomic atlas of gonadal sex determination

**Figure 1:**
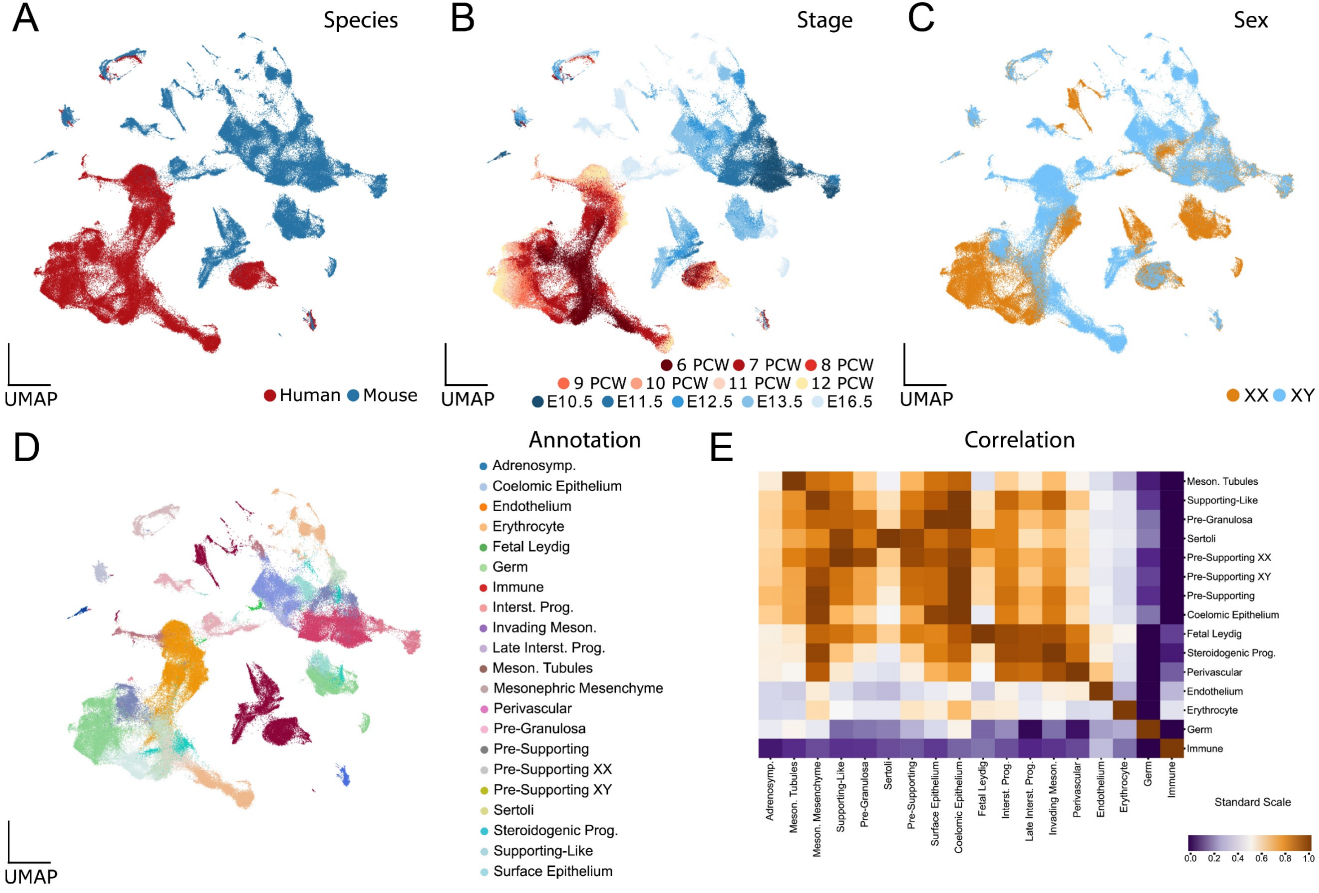
** Interspecies scRNA-seq data overlap: overview and analysis.** A to D) UMAP representations of the species-merged dataset, coloured by species (A), developmental stage (B), genetic sex (C) and annotated cell types (D). E) Correlation plot between the annotated cell types present in both species (mouse and human). Populations were ordered using hierarchical clustering based on correlation (Spearman) distance of expression levels between cell populations. A higher correlation value represents a higher inter-species similarity in a cell type, whereas a lower value represents a lower similarity.

To generate a comprehensive scRNA-seq atlas of human and mouse gonads, the two datasets were merged and compared using all the one-to-one ortholog genes. We then calculated the correlation coefficients between each human and mouse cell population and generated a UMAP to confirm that the overall structure of the data and the correlation between cell types was still maintained (**Figure 1E**, **Supplementary Figure S2**). The total number of cells after merging and filtering the human and mouse datasets was 193,609 cells, with 15,253 genes expressed in both datasets out of 16,568 possible one-to-one orthologs 66. This corresponds to the majority of the dataset, with 83.4% of the UMI in the human dataset and 81.9% in the mouse dataset being assigned to one-to-one orthologous genes (**Table 1**).

The UMAP of the human-mouse atlas shows a partial overlap between the different interspecific cell types (**Figure 1A**), while maintaining the temporal and sex patterns observed in the analyses of the individual data sets (**Figure 1B** and **C**). In particular, human and mouse cells of certain cell types are very close or even overlap, such as endothelial cells, erythrocytes, immune cells and, to a lesser extent, germ cells. In contrast, somatic cells of the gonad, such as those of the supporting or steroidogenic lineage, do not overlap (**Figure 1D**). To compare the transcriptional signature of distinct human cell types with their mouse counterparts, we used Spearman correlation analysis and hierarchical clustering (**Figure 1E**). This methodology enabled us to compare the transcriptome of each cell type of one species with each cell type of the other species, providing insights into their comparative similarity or divergence. These analyses revealed that cross-species transcriptomes of highly differentiated cells tended to show strong similarities, whereas those of poorly differentiated cell types, such as progenitors or differentiating cells, did not. For example, endothelial cells, erythrocytes, germ cells, immune cells, mesonephric cells, SCs and fetal Leydig cells show a high inter-species transcriptomic correlation, whereas low differentiated cell populations such as pre-supporting cells, SLCs, pre-granulosa cells, coelomic epithelial cells and steroidogenic progenitors show a lower inter-species correlation.

### Inter-species similarities and differences of the main cell types of the testis and ovary

To better characterize the interspecies similarities and differences of the main cell types of the testis and ovary, we performed three different types of analysis: Differential Expression Analysis (DEA), Gene Ontology analysis, and a ElasticNet regression analysis to determine the molecular signature of each cell type in the human and mouse models. In this study, our focus was mainly on the supporting cells (i.e. SCs, pre-granulosa cells and SLCs). Analyses for other cell types such as fetal Leydig cells and germ cells are described in **Supplementary Figures S3-4** and** Supplementary Table S2**.

### Human and mouse Sertoli cells display significant interspecies differences

To investigate the transcriptomic similarities and differences between mouse (E11.5-E16.5) and human (6-12 PCW) cells, we first assessed which differentially expressed genes (DEGs) were observed across the dataset by comparing each cell type to all other cell types (**Supplementary Table S2**). However, to reduce bias due to different developmental times between species, we performed a more focused analysis, comparing the development across the supporting lineage within each species, by comparing the pre-supporting cell clusters against the corresponding supporting cell types (i.e. SCs, pre-granulosa, SLCs). For this purpose, we retained only those genes whose mean log fold change (LogFC) was > 0.25 and whose P-value adjusted for false discovery rate was < 0.05.

**Figure 2:**
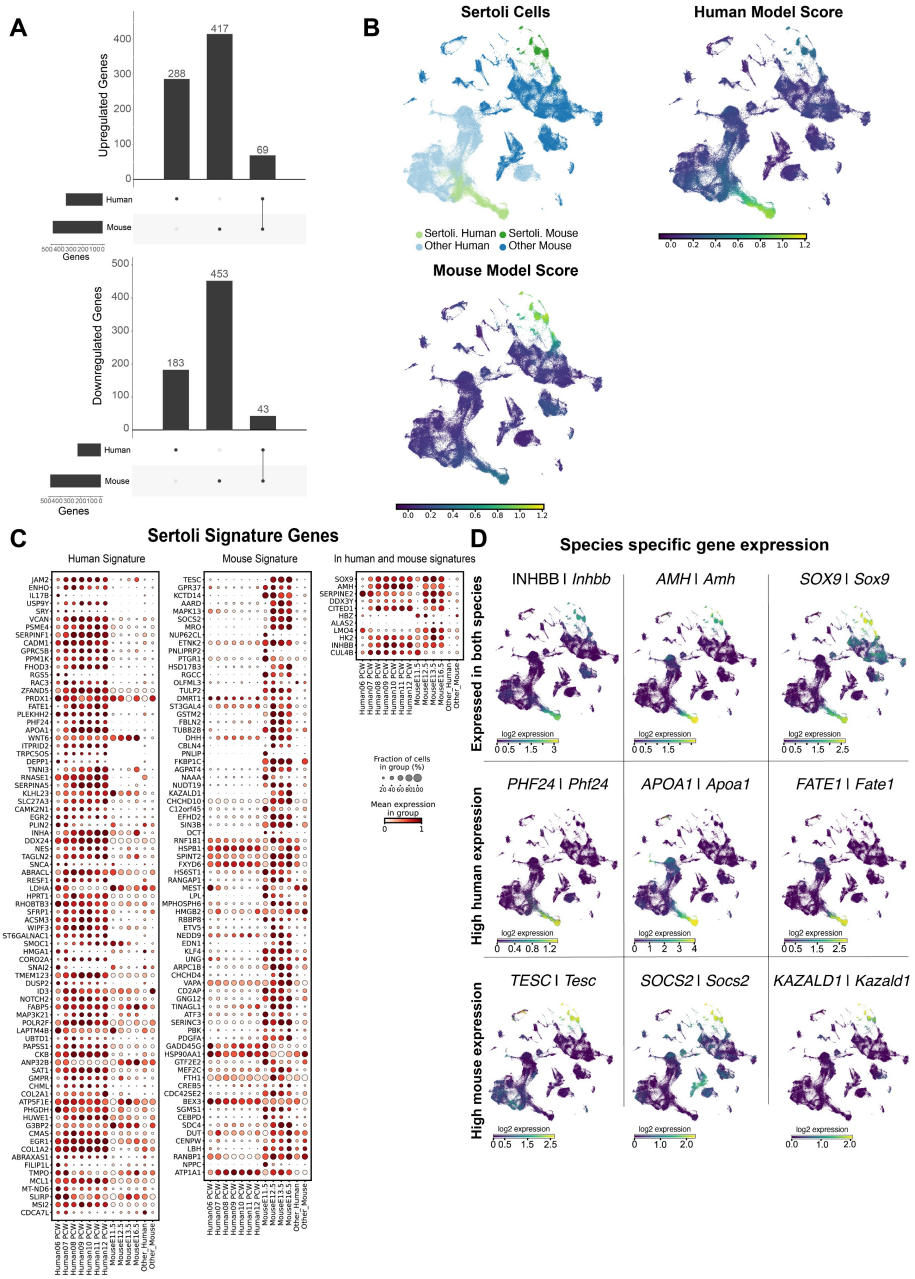
** Human and murine Sertoli cells (SCs) differ in overall gene expression.** A) Differentially expressed (DE) genes detected in SCs versus the pre-supporting clusters, per species**.** For up-regulated genes we found a total of 774 DE genes, of which 417 were exclusive to the human dataset, 288 were exclusive to the mouse dataset, and 69 were present in both datasets. For down-regulated genes, we found a total of 679 DE genes, 183 exclusive to the human dataset, 453 to the mouse dataset and 43 present in both. B) UMAP representation of the species-merged dataset, showing the location of the SCs in both species, and the ElasticNet regression model score based on the molecular signature of both mouse and human SCs. C) Dotplot representation positively weighted genes in each of the ElasticNet regression models (mouse SCs, human SCs) used to define SC transcriptomic signatures. Each dot represents gene expression within SCs at each developmental stage. The size of the dots reflects the proportion (in %) of SCs expressing the gene of interest and the colour reflects the level of expression. The "Other human" or "Other mouse" cell categories represent all other cell types present in the human or mouse dataset, respectively. D) UMAP representation, highlighting the expression of DE genes found to be common between both species, and species-specific genes.

Comparing the development across pre-supporting clusters to the SC clusters in both species, we found a total of 1,453 total differentially expressed genes, of which we had 112 genes (9%) in common between human and mouse SCs (**Figure 2A**). Of these, 69 were up-regulated and 43 were down-regulated in SCs compared to pre-supporting cells in both species. To better characterize the DEGs in SCs, we performed a functional term enrichment analysis to highlight the pathways (KEGG, Reactome), biological processes and molecular functions associated with them. We divided the analysis into three subsets: DEGs exclusive to the human dataset, DEGs exclusive to the mouse dataset and DEGs in both datasets. Genes in common to both species show enrichment for processes related to sexual differentiation (e.g. Transcriptional regulation of testis differentiation, development of primary male sex characteristics, development of primary sex characteristics, male sex differentiation, sex differentiation) (**Supplementary Table S11**). The DEGs exclusive to the human dataset show an enrichment for the pathways related to the development of the urogenital tract. Additionally, they show an enrichment for pathways related to the regulation of Insulin-like Growth Factor (IGF) (**Supplementary Table S12**). Finally, the DEGs exclusive to the mouse dataset show an enrichment for genes belonging to the WNT pathway; examining these genes and their expression, we found genes essential for SC differentiation, namely upregulation of *Fgf9* and downregulation of *Rspo1* (**Supplementary Table S13**).

To further characterize the transcriptomic profiles of human and mouse SCs, we used ElasticNet regression to determine a specific molecular signature for either human or mouse SCs compared to the rest of mouse or human cell populations. The aim is to identify sets of genes whose presence is characteristic of either human or mouse SCs or of both species. For convenience we called these genes “signature” genes. We have identified a set of 92 and 87 genes that define the genetic signature of human and mouse SCs, respectively. Of these genes, 81 and 76 are signature genes for human and mouse SCs respectively, while 11 are common to both species (see **Figure 2C**). Looking more closely at the expression profiles of these genes, only a small proportion are indeed common to both species. As expected, the genes *SOX9/Sox9*, *AMH/Amh*, *INHBB/Inhbb*, *DHH/Dhh, HK2/Hk2, CITED1/Cited1* are expressed in both human and mouse SCs and characterized as signature genes for both population of SCs (**Figure 2D**). However, the majority of SC-specific genes are highly expressed in either mice (e.g. *Aard, Socs2, Tesc, Hsd17b3*) or humans (e.g. *APOA1, FATE1, ENHO, CADM1*) (**Figure 2D**).

Overall, these results suggest that interspecies transcriptomic differences in SCs are substantial, while genes shared between the two species may represent a set of conserved genes that are potentially essential for SC specification and/or function.

### Human and mouse pre-granulosa cells display significant interspecies differences

**Figure 3:**
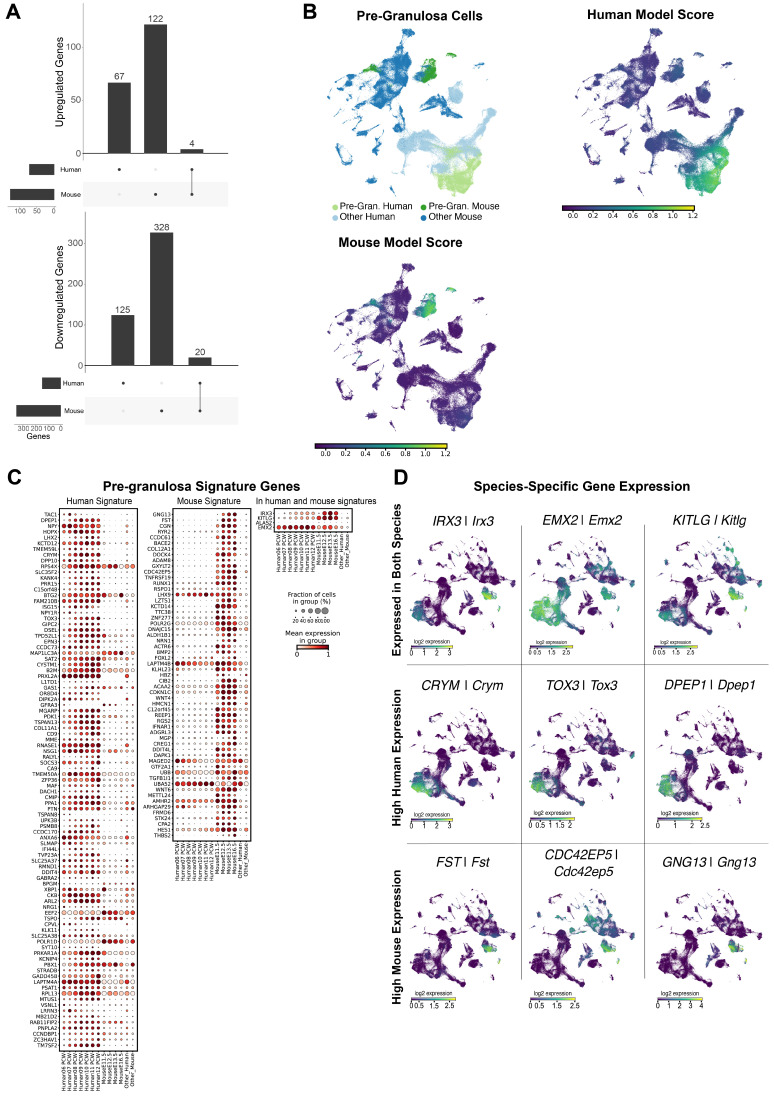
** Human and murine pre-granulosa cells differ in overall expression.** A) Differentially expressed genes detected in pre-granulosa cells versus the pre-supporting cell clusters, per species. For up-regulated genes we found a total of 193 DE genes, 67 of which exclusively in the human dataset, 122 exclusive to the mouse dataset, and 4 present in both datasets. For down-regulated genes, we found a total of 473 DE genes, 125 exclusive to the human dataset, 328 to the mouse dataset and 20 present in both. B) UMAP representation of the species-merged dataset, showing the location of the pre-granulosa cells in both species, and the ElasticNet regression model score for each training set. C) Dotplot representation of the positively weighted genes in each of the ElasticNet regression models used to predict the pre-granulosa cell location. Each dot represents gene expression within pre-granulosa cells at each developmental stage. The size of the dots reflects the proportion (in %) of pre-granulosa cells expressing the gene of interest and the colour reflects the level of expression. The "Other human" or "Other mouse" cell categories represent all other cell types present in the human or mouse dataset, respectively. D) UMAP representation, highlighting the gene expression of DE genes found to be common between species, and species-specific.

Using the same approach as for SCs, we investigated the transcriptomic similarities and differences between mouse (E11.5-E16.5) and human (6-12 PCW) pre-granulosa cells by comparing the pre-supporting cell clusters with the human and mouse pre-granulosa cells. Of the 666 DEGs in pre-granulosa cells, 24 are common to mouse and human, while 192 are human-specific and 450 are mouse-specific (**Figure 3A**). Similar to SCs, these results revealed large transcriptomic differences between human and mouse pre-granulosa cells. Functional term enrichment analysis revealed that the DEGs common to both species are enriched for processes related to gonadal development (e.g. female sex determination, sex determination, development of primary sexual characteristics) (**Supplementary Table S23**). Among the DEGs enriched in mice, we found genes related to WNT signalling pathways (*Igfbp2, Egr1, Cav1, App*, *Nkd1*, among others) as well as genes associated with the up-regulation of genes essential for granulosa differentiation, such as *Rspo1* and *Lgr5*. *Wnt4*, a gene essential for suppressing the development of supporting cells into SCs, is only upregulated in the mouse data set (**Supplementary Table S25**). Regarding specifically enriched DEGs in humans, we again found pathways related to the regulation of Insulin-like Growth Factor (**Supplementary Table S24**).

The ElasticNet regression identified 61 and 97 genes defining the mouse and human pre-granulosa gene signatures, respectively. Of these, 57 and 93 are signature genes for human and mouse pre-granulosa cells respectively, while 4 are common to both species (**Figure 3C**). Overall, the majority of the pre-granulosa signature genes are again either mouse-specific (e.g. *Fst, Gng13, Cdc42ep5*) or human-specific (e.g. *CRYM, TOX3, DPEP1*), with only few signature genes common to both species (*e*.*g*. *IRX3*/*Irx3*, *EMX2*/*Emx2*, *KITLG*/*Kitlg*) (**Figure 3D**). Taken together, these results reveal that the transcriptomic differences between mouse and human pre-granulosa cells are significant. These transcriptomic differences may reflect either different gene expression programs between the two species and/or differences in the differentiation status due to the different developmental rates between the two species.

### Human and mouse Supporting-like cells (SLCs) display also major interspecies differences

Recently, a population of SLCs was described in both humans and mice that express *PAX8/Pax8* as well as gonadal markers (*GATA4/Gata4, NR5A1/Nr5a1* and *WNT6/Wnt6*) 63, 67-70. They were named SLCs due to their transcriptomic similarities to the supporting cell lineage 63. These cells are specified very early at 6-8 PCW in humans and as early as E10.5 in mice and are located in the XX and XY genital ridge, along the border with the mesonephros. SLCs contribute to the formation of the rete testis and rete ovarii, and potentially to a significant proportion of the SC pool and pre-granulosa cells 63.

**Figure 4:**
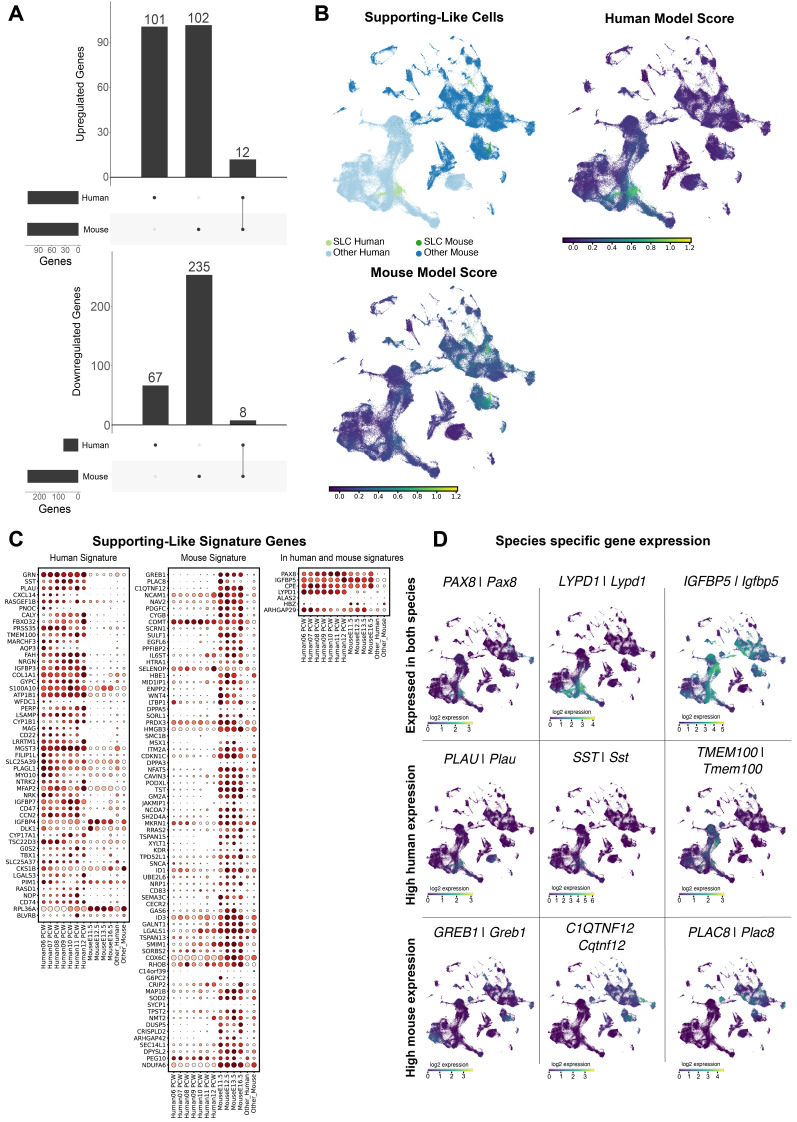
** Large differences in the expression of supporting-like cells (SLCs) between human and mouse.** A) Differentially expressed genes detected in SLCs versus the pre-supporting cell clusters, per species. For up-regulated genes we found a total of 115 DE genes, 101 of which exclusively in the human dataset, 103 exclusive to the mouse dataset, and 12 present in both datasets. For down-regulated genes, we found a total of 327 DE genes, 67 exclusive to the human dataset, 253 to the mouse dataset and 8 present in both. B) UMAP representation of the species-merged dataset, showing the location of the SLCs in both species, and the ElasticNet regression model score for each training set. C) Dotplot representation of the positively weighted genes in each of the ElasticNet regression models used to predict the SLC location. Each dot represents gene expression within SLCs at each developmental stage. The size of the dots reflects the proportion (in %) of SLCs expressing the gene of interest and the colour reflects the level of expression. The "Other human" or "Other mouse" cell categories represent all other cell types present in the human or mouse dataset, respectively. D) UMAP representation, highlighting the gene expression of DE genes found to be common between species, and species-specific.

Of the total 525 DE genes in mouse (E11.5-E16.5) and human (6-12 PCW) SLCs, only a small proportion of 3.8% (20 genes) are common between human and murine SLCs (**Figure 4A**). This reveals significant interspecies differences in SLC expression profiles. In the common set of genes, we found pathways relating to the transcriptional regulation of testis differentiation, regulation of Insulin-like Growth Factor, and WNT ligand biogenesis and trafficking (WNT6). Human-specific pathways show again regulation of Insulin-like Growth Factor. Mouse-specific pathways show an enrichment for NOTCH4 signalling pathways, PDGF signalling, among others (**Supplementary Table S35**) 71, 72.

In the ElasticNet regression models, we identified a set of 81 and 59 genes that define the gene signature of mouse and human SLCs, respectively. Of these genes, 52 and 74 are signature genes for human and mouse SLCs respectively, while 7 are common to both species (see **Figure 4C**). The vast majority of SLC-signature genes are indeed either mouse-enriched (e.g. *Greb1, Ciqtnf12, Podxl, Sulf1*) or human-enriched (e.g. *CXCL14, SST, PLAU, GRN, MGST3*) (**Figure 4D**). Among these, seven genes including *CPE/Cpe*, *IGFBP5/Igfbp5* and *PAX8/Pax8* - were found as signature genes common to both models. Taken together, these results suggest that the transcriptomic profiles of SLCs are highly divergent between mouse and human, with *PAX8/Pax8* as the major common signature marker.

**Figure 5:**
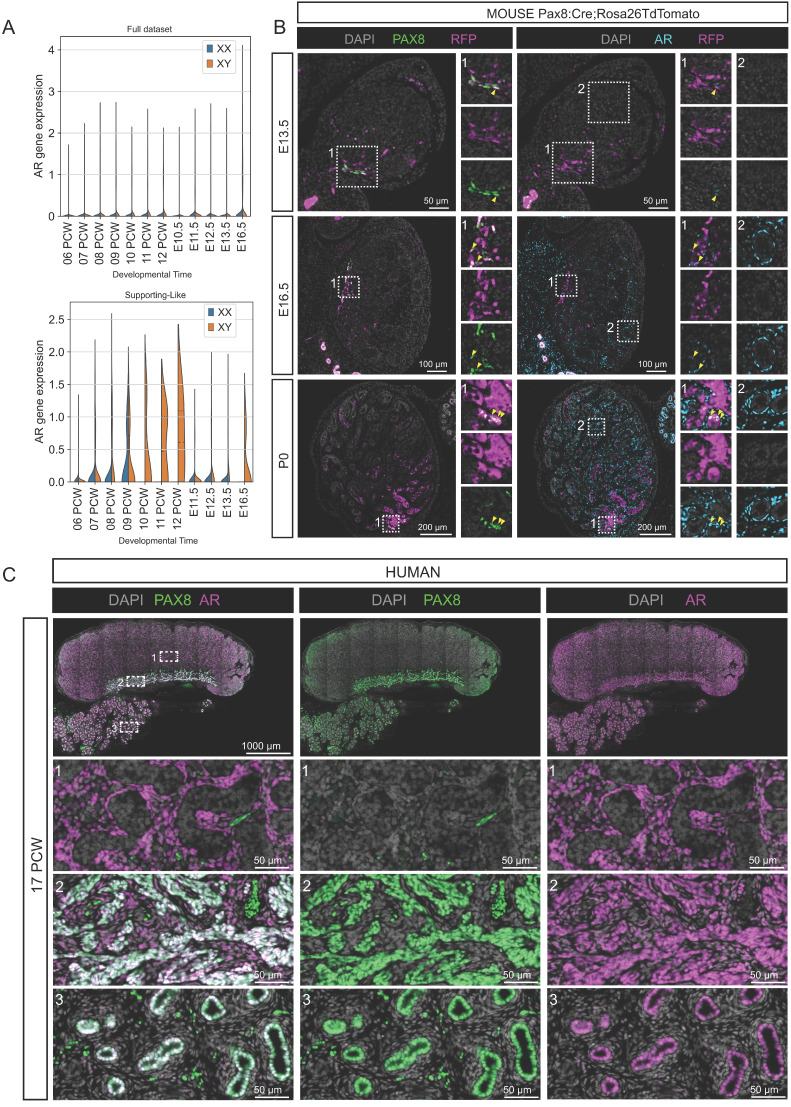
**AR is expressed in human but not in early mouse fetal SLCs**. (**A**) Violin plot representation of the expression of *AR*/*Ar* in both species over time. The top plot represents the overall expression of *AR*/*Ar* across all gonadal cells. The bottom plot represents the expression of *AR*/*Ar* in the SLCs. (**B**) Representative double immunofluorescence (IF) against PAX8 and RFP or AR and RFP on serial sections of *Pax8:Cre^ki/+^;Rosa26TdTomato^ki/ki^* mouse testis at E13.5, E16.5 and P0. Mouse SLCs express PAX8 and an increasing proportion co-express AR over time. Yellow arrowheads indicate cells co-expressing PAX8 and AR. At E13.5, few cells express AR, but all are located close to the rete testis. At E16.5, AR is widely expressed in the testicular interstitium (insets 2) and some cells of the rete co-express AR and PAX8 (insets 1). At P0, many but not all SLCs co-express PAX8 and AR. The insets are 100 µm wide. DAPI was used as a nuclear counterstain. (**C**) Representative double IF against PAX8 and AR in human testis at 17 PCW. Note that Human rete cells co-express PAX8 and AR at 17 PCW. The boxes show three regions magnified by enlarging the testis (1), the rete testis (2) and the epididymis (3). Note that AR is co-expressed with PAX8 in the SLCs of the rete testis, but is also expressed in the interstitial compartment and in the epithelium of the epididymal ducts. DAPI was used as a nuclear counterstain.

To illustrate these interspecific differences in expression, we focused on the androgen receptor (AR). We identified the *AR/Ar* gene as one of the genes differentially expressed between human and mouse SLCs (**Figure 5**). First, our scRNA-seq data show that *AR* is expressed early in the human gonad, as early as 9 PCW, and increases steadily in XY SLCs. In contrast, in the mouse, *Ar* expression in SLCs is low or absent between E10.5-E16.5 (**Figure 5A**). Second, to validate these results and to further characterize the spatio-temporal expression of AR in the developing testis and rete testis, we analyzed the expression of the SLC markers PAX8 and AR by immunofluorescence in developing mouse testis at E13.5, E16.5 and birth (P0) as well as in human fetal testis at PCW17 (**Figure 5B-C**). While AR is co-expressed with PAX8 in all human rete testis cells, the proportion of rete testis cells expressing AR in the mouse is initially very low at E13.5, this proportion gradually increases at E16.5 and then at P0, when the majority of cells in the rete testis co-express PAX8 and AR. Note that AR is not specific to rete testis and is also expressed in peritubular myoid cells and other interstitial progenitors as previously described 73, 74. These AR expression data therefore provide an example of differential expression between mice and humans, possibly as a result of differences in the paces of rete testis development.

## Discussion

For decades, the mouse has been the most widely used and preferred model organism for the study of mammalian gonadal sex determination and associated human diseases such as DSD, gonadal defects and infertility. The mouse is the model of choice because of its ease of use, the hundreds of mutant mouse strains currently available that mimic many disorders related to DSD and infertility, the ability to test functional hypotheses and the wealth of knowledge currently available. Mice and humans also share a similar genetic background, with approximately 90% of both genomes sharing regions of conserved synteny 75. But to what extent is the mouse a suitable model for human biology and more precisely can it be used to gain a better understanding of the human gonadal sex determination process and the diseases associated with it, such as DSD, gonadal abnormalities and infertility? Recently, the development of single-cell omics technologies has greatly advanced the field of comparative genomics, transcriptomics and epigenomics and can help us to understand the suitability of the mouse model for the study of human gonadal differentiation. Surprisingly, despite the increasing number of publications using single-cell RNA-seq and the characterisation of gonadal sex determination atlases in several species 4, 63, 67, 76, 77, very few have compared single-cell expression in humans and mice. Complications arise from the inherent difficulty of obtaining samples at comparable embryonic and fetal stages that have been technically processed and analysed in a similar way so that the data can be compared without technical bias. This is the strength of this study, as the generation of both atlases had been planned from the outset to be comparable and to minimise technical bias (see Material and Methods section). This included the use of the unique 10x Chromium Controller system to capture the cells and the version Single Cell 3′ Library & Gel Bead Kit to prepare the 3' libraries, a similar number of cells captured (around 100,000 cells per atlas) and a similar sequencing depth (between 100,000 and 150,000 reads per cell). Finally, the analyses were carried out using only the one-to-one orthologous genes, which allowed more than 80% of the UMIs in the human and mouse data sets to be retained, and the data were processed in an identical manner. The only potentially relevant differences are the time windows of the human and mouse data, the longer gestation period in humans and the asynchronous timing between the different cell types of different species. While the mouse single cell transcriptome atlas covers the entire process of sex determination and differentiation, from the emergence of the genital ridges at E10.5 to the fetal gonads at E16.5, the human atlas covers a slightly more limited period. Although all major cell types differentiate and appear in the human gonads between 6 and 12 PCW, this window does not include the early events of gonad formation that takes place at around 5 PCW, while the expression of *SRY* in the supporting progenitors initiates asynchronously just before 6 PCW 44.

Furthermore, unlike the mouse, where ovarian germ cells enter meiosis between E12.5 and E14.5 78-80, the human oogonia does not enter meiosis until 10 PCW 44, which is comparatively much later than in the mouse, but also very close to the late stages analysed in this study. Finally, in mice, which have a gestation period of only three weeks, the different cell types tend to differentiate synchronously in a short time (excluding pre-granulosa cells). In contrast, in humans, where gestation lasts nine months, asynchrony in the differentiation of different cell types is observed. For example, in germ cells, entry into meiosis is spread out over time. The first cells enter meiosis at around 10 PCW, a process that continues until 22 PCW. This is also the case for the temporal window of *SRY/Sry* expression in supporting progenitors of the gonad, which is very brief in mice but very spread out in time and asynchronous in humans 45. Nevertheless, we believe that a comparison of the major cell types of testis and ovary remains valid and necessary.

Three striking facts emerge from our interspecies comparisons between different testicular or ovarian cells. First, we observe large transcriptomic differences in each of the cell types present in the human and mouse gonads. Of the DE or cell type-specific genes, only a minority maintain a conserved expression profile between the two species. Secondly, we found a stronger correspondence between the transcriptomic signatures of more differentiated cell types in humans and mice. Thirdly, among the expression profiles that are conserved between humans and mice, we found genes that are known to be essential for the differentiation or function of gonadal cells in both species (*SOX9, AMH,* ...).

Another study by Garcia-Alonso *et al.* generated and compared scRNA-Seq atlases corresponding to mouse gonads between E10.5 and E12.5 and human gonads from the first and second trimesters of pregnancy (6-21 PCW) 67. Similar to our results, they found high similarity between the transcriptomic signatures of highly differentiated cell types such as endothelial, immune, germ, SC and Leydig cells. Cell types with low inter-species similarity were poorly differentiated cells such as mesenchymal and interstitial progenitors, but also pre-granulosa cells. The low similarity of pre-granulosa cells is likely to be a consequence of their somewhat weak expression program at the studied stages, their wave-like development and multiple origins, as well as potentially important developmental divergences between humans and mice 10, 81, 82. While the Garcia-Alonso study focused on the comparison of the differentiation programmes of a cell lineage, our study focused on a direct comparison of cell type to cell type, which makes the two studies rather complementary.

Garcia-Alonso *et al.* also identified two populations of early and late human SLCs that express *PAX8* in humans 67. This is consistent with our study of mouse SLCs, which shows that this lineage is specified from E10.5 and is initially sexually undifferentiated 63. From E12.5, sexual dimorphism appears with the progressive acquisition of SC and pre-granulosa-like profiles. In line with their findings, we have also identified SLCs in human gonads from PCW 6 64, the proportion of which decreases rapidly after PWC 8, particularly XX SLCs, consistent with the presence of a rudimentary rete ovarii at later stages 67. We also defined the specific molecular signature of human and mouse SLCs, as well as a signature common to both species. Our results are consistent with their analyses and show that the genes *PAX8, CXCL14, IGFBP5* are markers of SLCs common to both humans and mice. Our study also demonstrated the early expression of AR in SLCs of the fetal human rete testis (**Figure 5**). In contrast, in mice we observed a very low proportion of the rete testis expressing AR at E13.5, which gradually increases until birth. Our results are consistent with existing human data indicating that AR is expressed in the rete testis during the fetal, postnatal and adult periods 83-85. In contrast, in the rat, AR appears to be expressed in the rete testis only during the postnatal and adult periods 86-88. This raises the question of why there is such interspecific variation in AR expression and what the potential role of androgens might be in rete testis development and function, particularly in humans. It is possible that the differences between rodents and humans reflect differences in the pace of rete testis development or differentiation. To our knowledge, there are no data in mice or humans that suggest a role for AR and androgens in the development or function of the rete testis. Nevertheless, we believe that AR could be used in the future as a marker of human rete testis cells.

Overall, these results raise questions about the choice of the mouse as an animal model for studying human sex determination. In fact, these questions cannot be answered in a binary yes or no manner, but require further analysis of the cross-species transcriptomic profile of the cells under investigation. In particular, a conserved expression profile in both species seems to be a necessary condition. For example, in the case of SCs, the specific and conserved expression of genes such as *SOX9, AMH, INHBB* suggests that the mouse model is relevant for these transcription factors or signalling pathways. This is confirmed by similar phenotypes both in patients with pathogenic variants and in mutant mice null for these specific genes. Conversely, genes with a divergent expression profile in SCs, whether highly expressed in mice (such as *Aard, Tesc, Socs2*,...) or in humans (*PHF24, APOA1, FATE1*,...), question the relevance of the mouse model for these genes in particular. In the case of SLCs, for example, very few SLC-specific genes show similar expression profile between humans and mice, with rare exceptions such as *PAX8* and *IGFBP5*. The very different transcriptomic profiles of mouse and human SLCs that form the rete testis, an essential and highly conserved mammalian structure, raise the question of which genes or signalling pathways are responsible for their differentiation and function. More specifically, is a conserved gene such as *PAX8/Pax8* essential for the specification of SLCs? Similarly, are there divergent signalling pathways between mice and humans that are responsible for the differentiation and function of SLCs? Functional genomics studies in mice and the identification of pathogenic mutations in humans with DSD or azoospermia could help to answer these questions.

In summary, the question of whether the mouse is a good model for the study of human gonadal development and its pathologies is ill-posed and has no binary answer. It is therefore crucial to understand what part of mouse biology can be extrapolated to humans and which gene expression profiles or signalling pathways are conserved between the two species. Only genes with similar expression profiles in both species should be considered for the generation of transgenic mice as animal models if we want to gain insights into human biology and physiopathology. This condition is also crucial for the optimisation of animal model use and the reduction of the economic and ethical costs of animal research.

### Limitations of the study

Our cross-species analysis is based on gene expression. Often the level of expression of a gene does not correlate with its function and importance in a biological process. This raises the question of how relevant this analysis is for comparing a developmental process such as gonadal sex determination and whether the mouse is a good model for humans. Our analysis revealed a large variation in interspecific expression in the transcriptome of the main cells of the testis or ovary. Indeed, in each cell type, only a small fraction of genes have comparable expression profiles, often genes already known to have essential functions in gonadal sex determination (e.g. *Sox9/SOX9*, *Dhh/DHH*, ...). More specifically, it is difficult to draw conclusion from the comparison of germ cells, which have conserved transcriptomes but show a large number of differentially expressed genes. As mentioned above, meiosis entry starts at E12.5 in XX mouse and brings important changes in their transcriptome. Moreover, in mouse at E16.5, germ cells are nearly in dictyate while XX human germ cells at 12 PCW are only starting to enter prophase I of meiosis.

It is important to highlight some limitations of this study as well as biased interpretations of certain expression data. The first limitation is the large difference in the time required for processes such as gestation and gonadal sex determination; whereas these processes take weeks in humans, they take 24 to 48 hours in mice. It is also important to consider the asynchronous synchronisation between different cell types in different species, which can complicate analyses. In addition, cross-species comparisons of genes with large differences in expression levels give the false impression that the gene is not expressed in one of the species, especially when using UMAP plots. This is because the relative expression and scales used do not allow these low levels of expression to be visualised. In fact, gene expression is normalised depending on the type of visualisation. The colour coding of the expression is based on the expression range of the gene (the difference between minimum and maximum expression). If a gene has a narrower expression range in one condition or sample, it may not be possible to see the differences in expression in that sample.

## Methods

### Human tissue collection, single cell suspension and library preparation

Human embryos and fetuses aged 6 to 12 post-conception weeks (PCW) were obtained from legally induced terminations of pregnancy performed in Rennes University Hospital, in accordance with the legal procedure agreed by the National agency for biomedical research (declaration #PFS09-011; Agence de la Biomédecine) and the approval of the Local ethics committee of Rennes Hospital (advice # 11-48). Detailed procedures for the generation of single-cell RNA-seq data, including sample collection, were previously described 64. Briefly, the gonads were recovered from the aspiration products in ice-cold PBS and dissected free of mesonephros. Sexing was performed by morphological evaluation of the gonads or by qPCR for embryos younger than 7 PCW using previously published primers targeting the SRY 89 and GAPDH 90 loci. A total of 15 male and 15 female gonads were analysed, including 2 to 4 individuals of each sex at PCW 6, 7, 8, 9, 10 and 11-12. Single cell suspensions were obtained by a standard trypsin and mechanical digestion procedure. Approximately 4000 single cells per sample were captured using the Chromium™ Single Cell 3' Library & Gel Bead Kit v2 according to the manufacturer's instructions (10x Genomics). Sequencing was performed on an Illumina HiSeq 4000 instrument to reach a sequencing depth of at least 350 million paired-reads per sample.

### Dissection of mouse urogenital ridges and gonads, single cell suspension and library preparation

All animal procedures were performed in accordance to the ethical guidelines of the Service de la Consommation et des Affaires Vétérinaires (SCAV) of the Canton de Genève (experiment ID GE/57/18). Mouse embryos were collected at embryonic day (E)10.5, E11.5, E12.5, E13.5 and E16.5 from CD-1 outbred females mated with heterozygous Tg(Nr5a1-GFP) males as described in Mayere *et al.* 2022 63. Sexing was performed by PCR according to the protocol described in 91. Urogenital ridges at E10.5 and E11.5 and gonads at later time points were isolated for dissociation as previously described 76. Approximately 5000 single cells per sample were captured using 10x Chromium Controller. For each developmental stage sex combination, two captures from independent biological replicates were performed. Library were prepared with Single Cell 3′ Library & Gel Bead Kit v2 following manufacturer instructions 76. Sequencing was performed on Illumina HiSeq4000 in paired-end 26 + 98 + 8 bp mode with a targeted depth of 100 000 to 150 000 reads per cell at the Health 2030 Genome Center of Geneva as described in 76.

### Single-cell RNA sequencing analysis

#### Data pre-processing

Computational analysis was made partially at the Baobab HPC server at UNIGE. Demultiplexing, alignment and UMIs quantification were performed with the cellranger software suite (version 3.1, 10X Genomics). Protein coding genes and long non-coding RNAs were retained for further analysis. Gene-barcode matrices were generated with cellranger's count pipeline. Empty and low-quality barcodes were filtered out using a local minimum threshold on the UMIs *versus* barcode distribution of the raw matrices, as described in 76. Scanpy (version 1.6.0) python package was used for data normalization, annotation and visualization. Raw UMI counts were normalized for library size (normalize_per_cell function from Scanpy), log-transformed (log1p function from Scanpy) and normalized for sequencing depth (regress_out function on UMI count from Scanpy). For the downstream analysis, only genes detected in at least 3 cells, and cells expressing at least 50 genes were considered.

#### Dimensionality reduction, clustering and visualization

PCA was computed using 100 components (PCA function from Scanpy) on all genes expressed in more than 3 cells, and used as a basis for calculating the UMAP. For visualization, the corrected neighbourhood graph was embedded in two dimensions using UMAP 65 (umap function from Scanpy with default parameters). The same graph was also used for cell clustering via Leiden algorithm (Traag, *et al.*, 2019) (leiden function from Scanpy, resolution=1.5). Connections between clusters were estimated using PAGA (paga function from Scanpy with default parameters) 92.

#### Batch effect and cell cycle assessment and correction

Biases on cell cycle and batch effect were corrected using Harmony 93. To identify cell cycle bias, a list of genes associated to specific cell cycles was used to split them between G1, G2M and S 94.

#### Human transcriptomic atlas annotation

Differential gene expression for the human dataset was calculated between leiden clusters, with a Mann-Whitney-Wilcoxon test (FindAllMarkers function from Seurat R package - version 4.02) 95, 96, using MAST's Seurat wrapper function. Cell type annotation was done based on marker gene expression, developmental time points and sex. Marker genes were based on known literature, and the differential expression gene analysis results. For the cell lineage classification of the human leiden clusters, genes were considered to be unique to a cluster if expressed in over 50% of cells on a given cluster and at most 10% expression in all other clusters, with a maximum adjusted p-value of 0.05.

#### Human-mouse dataset merge

Human-mouse dataset merge was made by recovering all the one-to-one ortholog genes between mouse and human datasets from the Entrez database 97 and checking which were present on our dataset. From a total of 16,568 one-to-one ortholog genes, 15,253 were found expressed in both datasets. After filtering, a total of 340,746,090 human UMIs and 409,182,739 mouse UMI's were obtained, from which 284,282,256 and 335,021,579 were aligned to one-to-one orthologous genes, respectively. Harmony was used to assess and correct for species bias effect.

#### Human-mouse transcriptomic data analysis

Mouse cell annotation was based on the previous annotation described in 63. Differential gene expression for the merged dataset was calculated between the annotated cell types obtained in the respective individual species analyses, using the same approach previously described for the human dataset. Additionally, we sought to compare the progression of gene expression in supporting lineages between species in order to reduce for an interspecies temporal bias. For this we performed a DEA (logFC >0.25, adj_p value <0.05) between the pre-supporting cells and SCs, pre-granulosa cells and SLCs respectively, and compared the DE gene expression between species. Gene ontology classification was performed using Cluster Profiler for evidencing significant functional terms related to biological processes, molecular function, KEGG pathways, and Reactome pathways. Genes present on the enriched pathways were further detailed using STRING 98.

#### Interspecies cell type comparison

To determine what the most critical genes were to discriminate each cell type, we created a cell identity score training a ElasticNet model, with a one-versus-all approach. ElasticNet regression is a technique that weights the ability of each gene to classify a cell in a specific category - here the cell type of interest versus all other cell types. Compared to more ordinary regression technics such as least-square, ridge or logistic regression, ElasticNet outputs a model in which genes are attributed a weight of zero if they are inefficient for the classification. Cell signatures for each cell type are composed of the genes that have a non-zero positive weight. A gene that has a positive score does not mean that it has a critical biological role. It only means that it allows an efficient classification of the cell. The ElasticNet models were generated with two separate datasets with all genes expressed in more than 3 cells in input: the human dataset and the mouse dataset. In order to reduce the effects of unbalanced cell frequency across cell types (ie: overrepresentation), we randomly sampled 1000 cells per cell type in order to create the training sets. Fitted models were then used to score the cell identity on the human-mouse merged dataset 99.

### Human sample collection and immunofluorescence

Human fetal testes with attached mesonephroi (17PCW) were obtained from elective abortions (no medical indication) and donated for scientific research with written informed consent. Ethical approval was obtained from the Medical Ethics Committee of the Leiden University Medical Centre (B21.052). Developmental age was estimated by obstetric ultrasonography before the procedure. The isolated material was fixed in 4% paraformaldehyde overnight at 4°C, embedded in paraffin, sectioned (5-µm thick sections) and processed for immunofluorescence as previously described 100. Antigen retrieval was performed using TRIS-EDTA pH 9.0 (10 mM TRIS and 1 mM EDTA) for 20 minutes at 98°C. After cooling, the sections were treated with blocking solution of 1% bovine serum albumin (BSA) (Sigma-Aldrich) in PBST (0.05% Tween-20 in PBS) for 1 hour at room temperature, followed by incubation with primary antibodies (rabbit anti-PAX8, 1:1000, ProteinTech Group, 10336-1-AP; mouse anti-AR, 1:100, Santa Cruz Biotechnology, sc-7305) diluted in blocking solution overnight at 4°C. The sections were then washed twice in PBS and once in PBST and incubated with secondary antibodies (donkey anti-rabbit 488, Life Technologies, A21206; donkey anti-mouse 647, Life Technologies, A31571) diluted in blocking solution for 1 hour at room temperature. DAPI (1:1000, Life Technologies, D3571) was used to stain cell nuclei. Immunofluorescence images were captured using a ZEISS Axioscan 7 slide scanner (ZEISS). Acquired images were assembled using Adobe Illustrator 2021.

### Animals, sample collection and mouse immunofluorescence

Animals were housed and cared according to the ethical guidelines of the Service de la Consommation et des Affaires Vétérinaires (SCAV) of the Canton de Genève (experimentation ID GE35). Embryos from timed matings (day of vaginal plug = E0.5) were collected and fixed overnight at 4°C in 4% paraformaldehyde, serially dehydrated, embedded in paraffin, and 5-μm-thick transverse sections were prepared. After rehydration, sections were blocked for 2 hours at room temperature. Primary antibodies were incubated overnight at 4°C (rabbit anti-PAX8 1:200, Cell Signaling Technologies, #59018; rabbit anti-AR 1:100, Santa Cruz Biotechnology, sc-816; mouse anti-RFP (dsRed) 1:500, Santa Cruz Biotechnology, sc-390903). Secondary antibodies were incubated 1 hour at room temperature (chicken anti-rabbit 647, Thermo Fischer Scientific, A21443; donkey anti-mouse 555, Thermo Fischer Scientific, A31570). DAPI (1:1000) was used as nuclear counterstain. Fluorescence images were acquired using an Axio Imager M2 or Z1 microscope (ZEISS, Germany) fitted with an Axiocam 702 mono camera or MRm camera (ZEISS, Germany). Images were minimally processed for global levels with ZEN (ZEISS, Germany).

### Data and code availability

Raw human sequencing data and the corresponding gene count matrix data are available at the European Genome-Phenome Archive (EGA) under the accession number EGAS00001006568. The mouse scRNA-seq datasets are available at NCBI Gene Expression Omnibus GEO: GSE184708.

All code used for analysis is available upon request.

### Web interface

Both human and mouse gonadal gene expression data are included in ReproGenomics Viewer 101, 102.

This human and mouse combined gonadal atlas is freely accessible through a CellXgene interactive web portal (https://www.unige.ch/medecine/nef/datasets/) allowing one to query for genes of interest per cell type and developmental stage.

## Supplementary Material

Supplementary figures and table legends.

Supplementary table 1.

Supplementary table 2.

Supplementary tables 3-10.

Supplementary tables 11-46.

## Figures and Tables

**Table 1:** Total number of UMI recovered per dataset, separated by the how many of them are attributed to one-to-one orthologous genes versus other, which include one-to-many orthologs, many-to-many orthologs, and genes with no known orthologs.

	Human	Mouse
Total UMI count	340,746,090	409,182,739
UMI aligning to one-to-one orthologous genes	284,282,256	335,021,579
Other UMI	56,463,834	74,161,160
% of total UMIs kept	83.4%	81.9%
