# Supplementary material for: How relevant is the mouse model for understanding human sex determination?
Source: Int J Biol Sci. 2025 Oct 15;21(15):6599–615. doi: 10.7150/ijbs.90231 (PMC12631067; doi:10.7150/ijbs.90231)

Data Files

Data Files 1 – Coefficient scores obtained with the ElasticNet models evidencing the most efficient genes in characterizing a specific cell type (signature genes), using three distinct datasets (human data-only, mouse data-only), per cell.

Supplementary Figures

Supplementary Figure S1. Human scRNA-seq data overview and analysis. A) Representation of the data used to perform the analysis. 30 libraries, evenly split by sex, divided between seven distinct developmental stages: 6 to 12 post-conception weeks (PCW). Total cell count was of ~100.000 cells. B to D) UMAP representation of the human dataset, colored by sex (B), time points (C) and leiden clusters (D). E) UMAP and barplot representation of the human dataset, highlighting the annotated cell types and their overall abundance over time and sex. F) Dotplot representation of major cell markers in each of the populations.

Supplementary Figure S2 – UMAP representation of the human and mouse datasets using only the one-to-one orthologous genes. A-C) UMAP representation of the human and mouse datasets, coloured by sex (A), time points (B), and the annotated cell types (C).

Supplementary Figure S3 – Human and murine Leydig cells differ in overall expression. Differentially expressed genes detected in Leydig cells versus the steroidogenic progenitor/interstitial progenitor (human and mouse, respectively) cell clusters. For up-regulated genes we found a total of 700 DE genes, 144 of which exclusively in the human dataset, 465 exclusive to the mouse dataset, and 91 present in both datasets. For down-regulated genes, we found a total of 520 DE genes, 132 exclusive to the human dataset, 282 to the mouse dataset and 106 present in both. B) UMAP representation of the species-merged dataset, showing the location of the Leydig cells in both species, and the ElasticNet regression model score for each training set. C) Dotplot representation of the positively weighted genes in each of the ElasticNet regression models used to predict the Leydig cell location. Each dot represents gene expression within Leydig cells at each developmental stage. The size of the dots reflects the proportion (in %) of SCs expressing the gene of interest and the colour reflects the level of expression. The "Other human" or "Other mouse" cell categories represent all other cell types present in the human or mouse dataset, respectively. D) UMAP representation, highlighting the gene expression of DE genes found to be common between species, and species-specific.

Supplementary Figure S4 – Human and murine germ cells differ in overall expression. Differentially expressed genes detected in germ cells versus all the other cell clusters, per

species. For up-regulated genes we found a total of 2758 DE genes, 846 of which exclusively in the human dataset, 1645 exclusive to the mouse dataset, and 267 present in both datasets. For down-regulated genes, we found a total of 2950 DE genes, 1140 exclusive to the human dataset, 796 to the mouse dataset and 1014 present in both. B) UMAP representation of the species-merged dataset, showing the location of the Germ cells in both species, and the ElasticNet regression model score for each training set. C) Dotplot representation of the positively weighted genes in each of the ElasticNet regression models used to predict the Germ cell location. Each dot represents gene expression within SCs at each developmental stage. The size of the dots reflects the proportion (in %) of germ cells expressing the gene of interest and the colour reflects the level of expression. The "Other human" or "Other mouse" cell categories represent all other cell types present in the human or mouse dataset, respectively. D) UMAP representation, highlighting the DE gene expression of genes found to be common between species, and species-specific.

Supplementary Tables

Supplementary Table S1 – Human DEA based on the comparison of gene expression between Leiden clusters.

Supplementary Table S2 – Human versus Mouse DEA analysis based on all annotated cell types

Supplementary Table S3 – Human Sertoli versus Pre-supporting DEA based on the 1-to-1 gene dataset obtained from the interspecies overlap

Supplementary Table S4 – Mouse Sertoli versus Pre-supporting DEA based on the 1-to-1 gene dataset obtained from the interspecies overlap

Supplementary Table S5 – Human pre-granulosa versus Pre-supporting DEA based on the 1-to-1 gene dataset obtained from the interspecies overlap

Supplementary Table S6 – Mouse pre-granulosa versus Pre-supporting DEA based on the 1-to-1 gene dataset obtained from the interspecies overlap

Supplementary Table S7 – Human SLC versus Pre-supporting DEA based on the 1-to-1 gene dataset obtained from the interspecies overlap

Supplementary Table S8 – Mouse SLC versus Pre-supporting DEA based on the 1-to-1 gene dataset obtained from the interspecies overlap

Supplementary Table S9 – Human Leydig versus Steroidogenic Progenitor DEA based on the 1-to-1 gene dataset obtained from the interspecies overlap

Supplementary Table S10 – Mouse Leydig versus Interstitial Progenitor DEA based on the 1-to-1 gene dataset obtained from the interspecies overlap

Supplementary Tables S11-46 – Gene Ontology Enrichment Analysis results (Biological Processes, Molecular Function, KEGG pathways and Reactome pathways) for Sertoli Cells (**S11-22**), pre-granulosa cells (**S23-34**), and SLCs (**S35-46**) for human-specific DEGs, mouse-specific DEGs, and DEGs in common between species.

Supplementary Figure 1

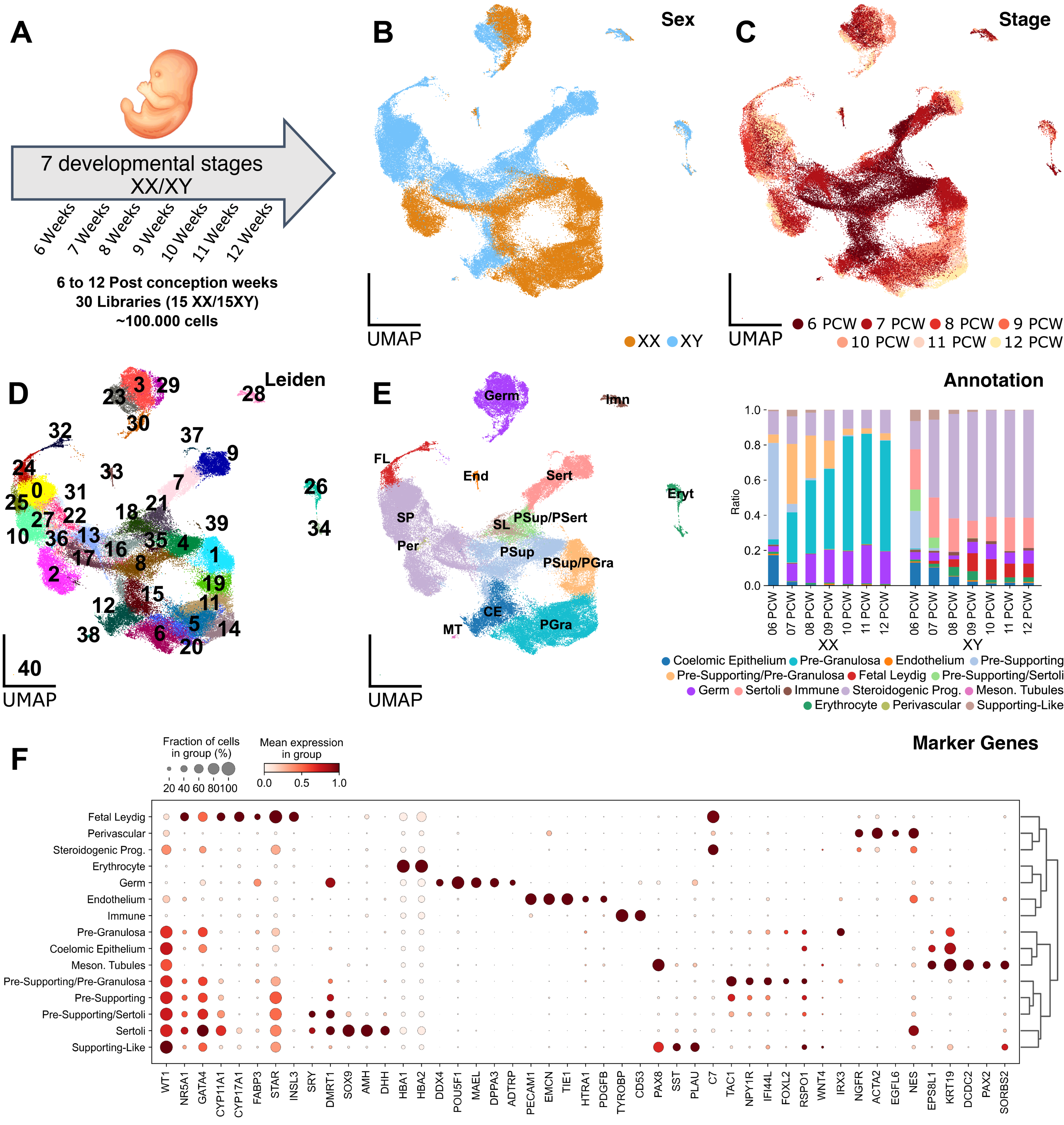

Supplementary Figure 2

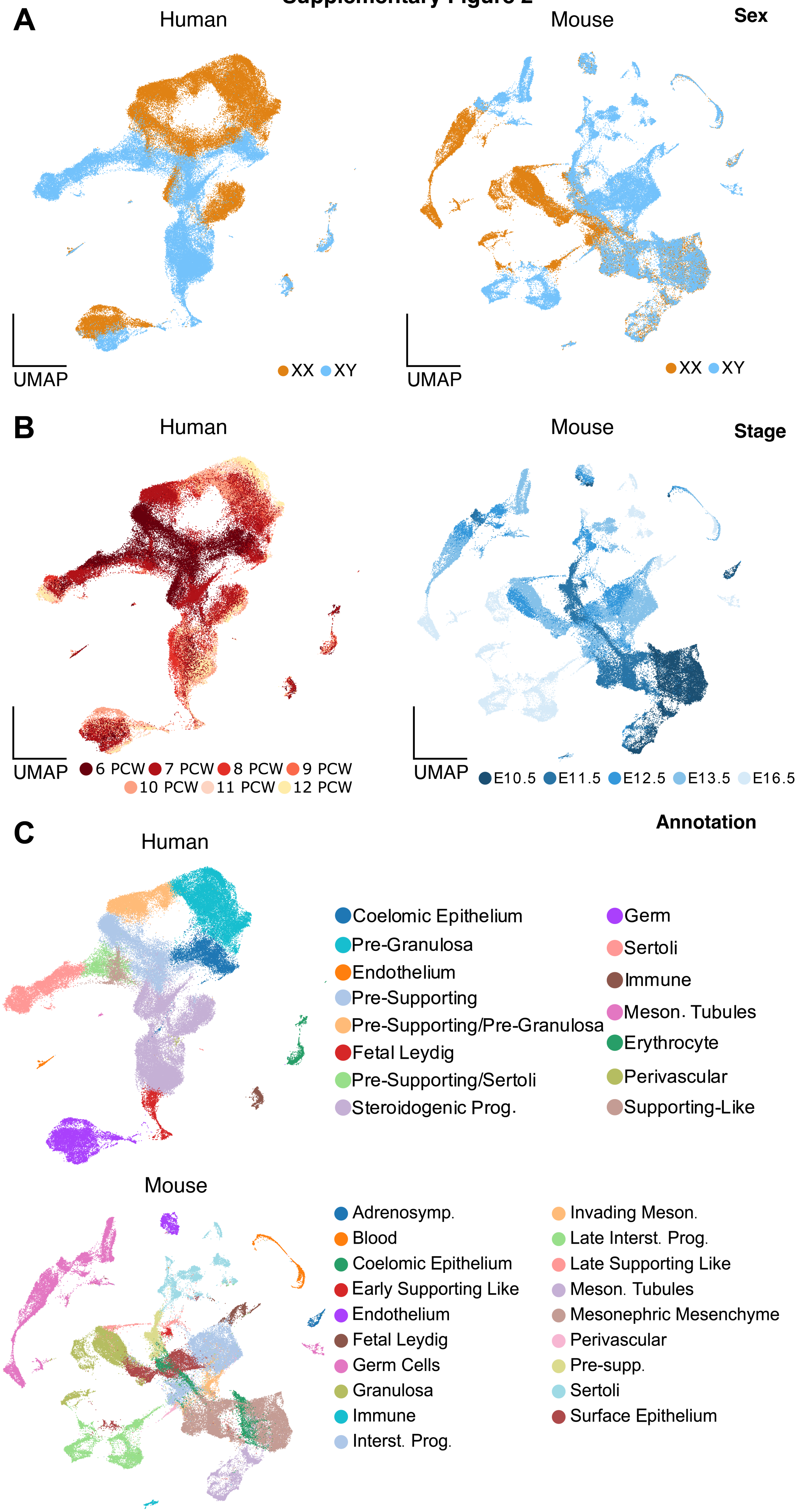

Supplementary Figure 3

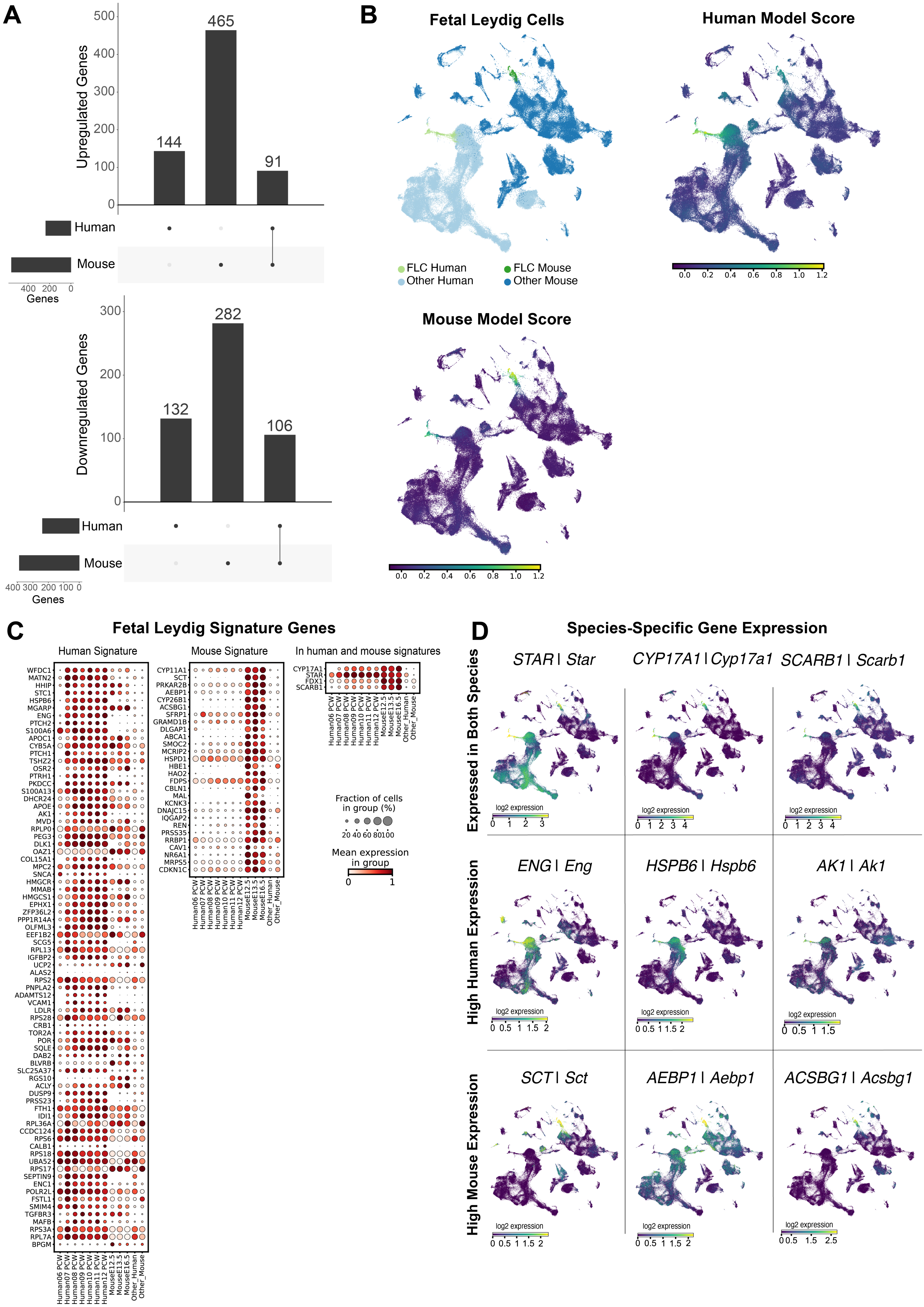

Supplementary Figure 4

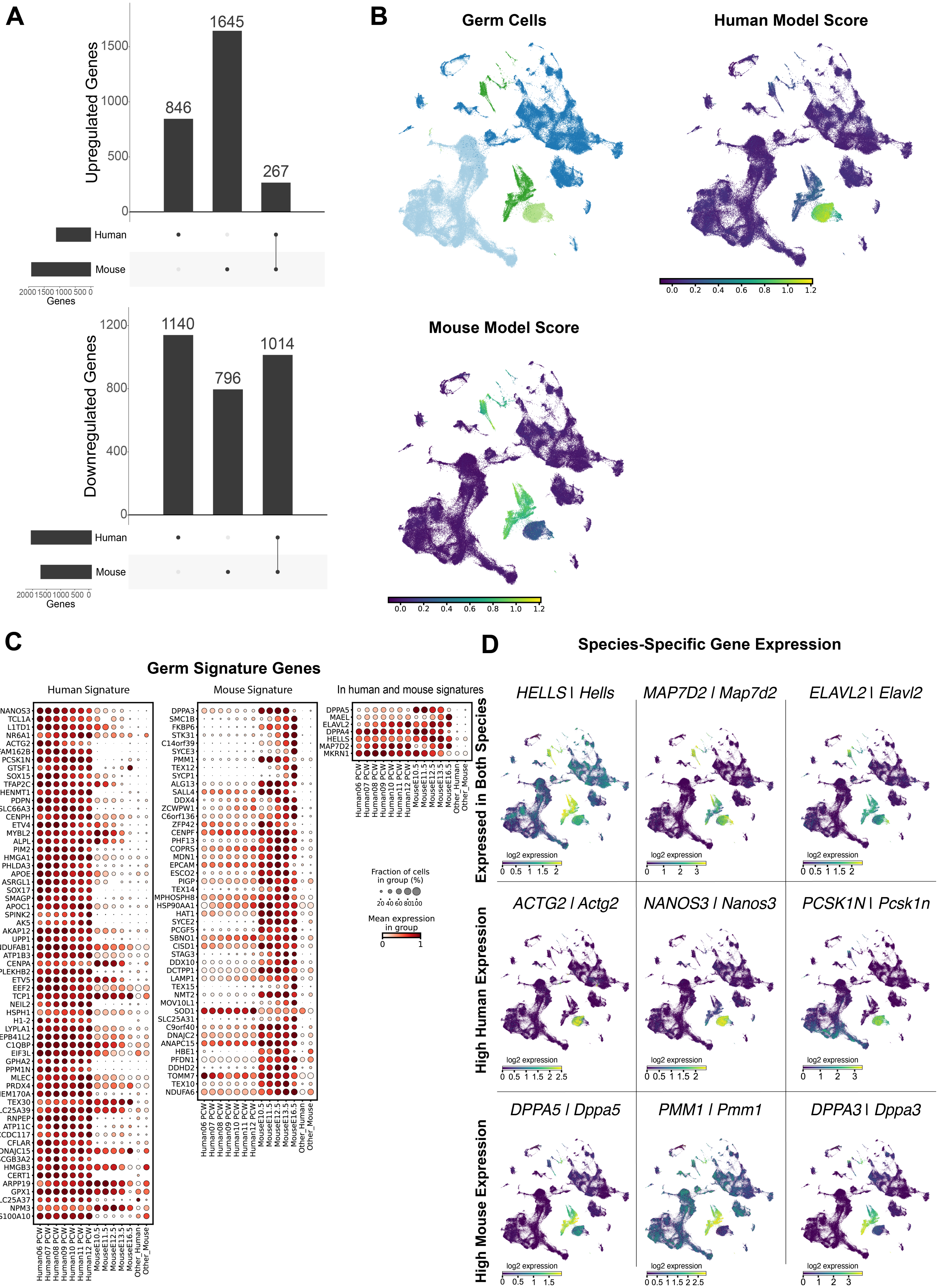

Supplement: Supplementary file 1 — Supplementary figures and table legends. [file ijbsv21p6599s1.pdf]
